# Distinct gene expression profiles associated with Notch ligands Delta-like 4 and Jagged1 in plaque material from peripheral artery disease patients: a pilot study

**DOI:** 10.1186/s12967-017-1199-3

**Published:** 2017-05-04

**Authors:** Giorgio Aquila, Cinzia Fortini, Antonio Pannuti, Serena Delbue, Micaela Pannella, Marco Bruno Morelli, Cristiana Caliceti, Fausto Castriota, Monica de Mattei, Alessia Ongaro, Agnese Pellati, Pasquale Ferrante, Lucio Miele, Luigi Tavazzi, Roberto Ferrari, Paola Rizzo, Alberto Cremonesi

**Affiliations:** 1 Department of Medical Sciences, University of Ferrara, Ferrara, Italy; 2 Department of Genetics and Stanley Scott Cancer Center, Louisiana State University Health Sciences Center and Louisiana Cancer Research Consortium, New Orleans, LA USA; 3 Department of Biomedical, Surgical and Dental SciencesUniversity of Milan, Milan, Italy; 4 GoldyneSavad Institute of Gene Therapy, Hadassah-Hebrew University Medical Center, 91120 Jerusalem, Israel; 5 IRCCS Neuromed, Angio-Cardio-Neurology Department, Pozzilli, Italy; 6 Department of Chemistry “G. Ciamician”, University of Bologna, Bologna, Italy; 7 Maria Cecilia Hospital, GVM Care & Research, E.S. Health Science Foundation, Cotignola, Italy; 8 Department of Morphology, Surgery and Experimental Medicine, University of Ferrara, Via Fossato di Mortara 64/B, 44121 Ferrara, Italy

**Keywords:** Peripheral artery disease, Notch, Inflammation, Vascular smooth muscle cells, Macrophages, microRNA

## Abstract

**Background:**

The lack of early diagnosis, progression markers and effective pharmacological treatment has dramatic unfavourable effects on clinical outcomes in patients with peripheral artery disease (PAD). Addressing these issues will require dissecting the molecular mechanisms underlying this disease. We sought to characterize the Notch signaling and atherosclerosis relevant markers in lesions from femoral arteries of symptomatic PAD patients.

**Methods:**

Plaque material from the common femoral, superficial femoral or popliteal arteries of 20 patients was removed by directional atherectomy. RNA was obtained from 9 out of 20 samples and analysed by quantitative reverse transcriptase-polymerase chain reaction (qRT-PCR).

**Results:**

We detected expression of Notch ligands Delta-like 4 (Dll4) and Jagged1 (Jag1), of Notch target genes Hes1, Hey1, Hey2, HeyL and of markers of plaque inflammation and stability such as vascular cell adhesion molecule 1 (VCAM1), smooth muscle 22 (SM22), cyclooxygenase 2 (COX2), Bcl2, CD68 and miRNAs 21-5p, 125a-5p, 126-5p,146-5p, 155-5p, 424-5p. We found an “inflamed plaque” gene expression profile characterized by high Dll4 associated to medium/high CD68, COX2, VCAM1, Hes1, miR126-5p, miR146a-5p, miR155-5p, miR424-5p and low Jag1, SM22, Bcl2, Hey2, HeyL, miR125a-5p (2/9 patients) and a “stable plaque” profile characterized by high Jag1 associated to medium/high Hey2, HeyL, SM22, Bcl2, miR125a and low Dll4, CD68, COX2, VCAM1, miR126-5p, miR146a-5p, miR155-5p, miR424-5p (3/9 patients). The remaining patients (4/9) showed a plaque profile with intermediate characteristics.

**Conclusions:**

This study reveals the existence of a gene signature associated to Notch activation by specific ligands that could be predictive of PAD progression.

**Electronic supplementary material:**

The online version of this article (doi:10.1186/s12967-017-1199-3) contains supplementary material, which is available to authorized users.

## Background

Most peripheral artery disease (PAD patients) are diagnosed at advanced stages of the disease when the arteries are already seriously compromised [1]. Beside medical treatment, current therapeutic approaches for symptomatic PAD consist of revascularization with angioplasty and stenting, atherectomy or bypass grafting. However, restenosis is a serious complication occurring in about 2/3 of the patients undergoing revascularization [2–4]. Thus, restenosis after endovascular treatment of atherosclerotic lesions remains a challenging clinical problem [4] and understanding factors that contribute to its pathophysiology could help predicting occlusion recurrence and/or interfering with it.

Atherosclerotic plaques are characterized by macrophage infiltration and abnormal proliferation of vascular smooth muscle cells (VSMCs) migrated from the medial to the intimal layer. Under the action of pro-inflammatory cytokines released by infiltrating macrophages, intra-plaques VSMCs transdifferentiate from a quiescent-contractile to a secretory-proliferative phenotype. This phenotype is characterized by expression of vascular cells adhesion molecules and inflammatory enzymes such as cyclooxygenase-2 (COX2), and contributes to the progression of atherosclerosis (reviewed in [5]). Since plaques prone to rupture contain a lower proportion of VSMCs compared to stable plaques, VSMCs survival is a major determinant of plaque stability [6] and proliferation of VSMCs plays a major role in in-stent restenosis [7].

The Notch signaling pathway is a major regulator of VSMCs and macrophages functions [8, 9]. This pathway is mediated by four transmembrane receptors (Notch1-4) and five ligands (Delta like 1, 3, 4, Jagged 1, 2). Ligands expressed on the surface of adjacent cells activate Notch receptors, triggering two proteolytic cuts that generate the active form of the receptor, NotchIC. The latter translocates to the nucleus where it binds transcriptional factor CSL (CBF-1, Suppressor of Hairless and Lag-1) thus promoting the transcription of Notch target genes. Established Notch target genes belong to the Hes (hairy/enhancer of split 1) and Hey (Hes-related proteins) families, involved in the transcription of downstream genes that can either maintain cell in an uncommitted state or induce differentiation [10, 11]. Different Notch receptors have distinct sets of target genes [12]. Moreover, activation of Notch receptors by different ligands can trigger different responses [13–17]. Jagged1 (Jag1)—mediated Notch activation is required for the expression of contractility marker smooth muscle (SM) 22 in VSMCs [18–21]. Activation of Notch1 and 3 prevents VSMCs apoptosis [22] and it counteracts their trans-differentiation from a quiescent/contractile to a proliferative/secretory phenotype promoted by interleukin (IL) 1-β [23, 24]. Differently from VSMCs, activation of Notch signaling promotes inflammatory response in macrophages [25] and leads to M1 (pro-inflammatory) rather than M2 (anti-inflammatory) gene expression by activation of NF-kappa B [26] and by reprogramming mitochondrial metabolism [27]. Delta-like ligand 4 (Dll4)-Notch3 signaling has been shown to mediate the response of macrophages to pro-inflammatory stimuli [28, 29]. These data suggest an involvement of the Notch signaling in determining the development and evolution of plaques in PAD patients.

In addition, several microRNAs (miRs) have been identified which play a role in atherosclerosis (reviewed in [30]). Endothelial cells-secreted miR126-5p promotes VSMCs turnover [31] and miR424/322 is involved in restenosis in injured rat carotid arteries [32] and in plaque rupture in mice [33]. miR21-5p and 146-5p promote VSMCs proliferation [34] and miR21-5p has been associated to plaque instability [35, 36]. miR155-5p plays a key role in atherogenic programming of macrophages to sustain and enhance vascular inflammation [37]. On the contrary, miR125a-5p has a protective effect in atherosclerosis since it decreases the production of inflammatory cytokines in oxLDL (oxidized low density lipoproteins) -stimulated monocyte-derived macrophages [38] and promotes the anti-inflammatory macrophage phenotype M2 [37]. Cross-talks between some of these miRs (miR126a-5p, miR21-5p, miR155-5p) and the Notch pathway have been described [34, 39–41].

The aim of this pilot study was the characterization of Notch signaling in plaque material from atherosclerotic lesions of PAD patients in relation to known markers of inflammations and plaque stability. We focused on the relative quantitation of mRNAs for Notch pathway components (Delta-like ligand 4, Jagged1 and target genes Hes1, Hey1, 2, L) and for markers of inflammation and VSMCs trans-differentiation and survival (CD68, COX2, Bcl2, SM22, miRs21-5p, 125a-5p, 126-5p, 146-5p, 155-5p, 424-5p). The relationship between these variables will be discussed.

## Methods

### Subject selection and interventional procedure

Twenty patients with documented ischemic, symptomatic lower extremities artery disease referred to our Institution with clinical indication for endovascular treatment were consecutively enrolled. Approval from the Ethics Committee of Maria Cecilia Hospital-Cotignola-Ravenna and written informed consent from patients were obtained according to the World Medical Association Declaration of Helsinki. Patients underwent a complete clinical assessment, including 6-min walk test, Doppler ultrasound examination and a pre-procedural angiography. Target lesion consisted of a *de novo*, single solitary lesion with diameter stenosis greater than or equal to 70% and cumulative lesion length greater than or equal to 15 cm. A 6 and 12 month follow-up was planned. Plaque material from the common femoral, superficial femoral or popliteal arteries was removed by rotational atherectomy followed by balloon dilatation. A 7-F sheath (Avanti, Cordis; or Balkin, Cook, Bjaeverskov, Denmark) that is compatible with a monorail-guided atherectomy catheter (Silverhawk P4010 or P4011 debulking catheter, FoxHollow) was used. The number of lesion passes was left to the discretion of the interventionist. The material obtained was dissected in 50 mm segments [42] and pools of fragments representative of each pass were freshly frozen in liquid nitrogen and stored at −80 °C or formalin-fixed/paraffin embedded.

### Histological and immunohistochemical analyses

5 μm sections were cut from formalin-fixed, paraffin-embedded samples and stained with hematoxylin-eosin and Masson’s trichrome according to routine histologic protocols. Plaque material was characterized for cellularity, inflammatory cells, fibrous tissue, calcification and lipids content, as previously described for coronary arteries plaques [43]. Frozen tissues were embedded in optimal cutting temperature compound (OCT) and cut into 10 μm sections for immunohistochemical analysis. Expression of macrophage marker CD68, smooth muscle actin (α-SMA), Notch receptors 1, 3 and ligands Delta-like 4 (Dll4) and Jagged1 (Jag1) was assessed by immunohistochemistry using a commercially available kit and following the manufacturer’s instruction (Vectastain ABC kit, Vector Laboratories, Burlingame, CA, USA). Primary antibodies were: rabbit anti-human Val1744-cleaved Notch1 (Cell Signaling #2421, Danvers, MA, USA, dilution 1:50), rabbit anti-human Notch1 (Santa Cruz, sc-6014, clone C20, Santa Cruz, CA, USA, dilution 1:100), rabbit anti-human Notch3 (Abcam, ab23426 m, Cambridge, UK, dilution 1:300), rabbit anti human α-smooth muscle actin (Novus Biologicals, NB600-531, Littleton, CO, USA, dilution 1:200), mouse anti-human CD68 (Dako M0814, clone KP1, Glostrup, DK, dilution 1:100). Primary antibody incubation was performed at room temperature for 1 h. Negative controls were sections to which only secondary antibody was added. Slides were scanned by Aperio Scan-Scope Digital Slides Scanner (Leica Biosystems, Buffalo Grove, IL, USA) and the percent of positive area was analyzed using ImageScope v11.1.2.

### Rat aortic smooth muscle cells isolation and culture

Rat aortic smooth muscle cells (RASMCs) were obtained from the thoracic aortas of 2 month-old Winstar rats, as described [44]. Experiments were performed in adherence with the Institutional Guidelines on the Use of Laboratory Animals. Cells were grown at 37 °C with 5% CO_2_ in DMEM containing 10% FBS, 100 units/ml penicillin, and 100 μg/ml streptomycin. To confirm the identity of isolated cells, immunofluorescent staining against α-SM-actin was performed. Cells were fixed in methanol and blocked in PBS 1X + 3% BSA and incubated with α smooth muscle actin primary antibody (Novus Biologicals, NB600-531, Littleton, CO, USA, dilution 1:200) for 1 h at room temperature followed by immunoreaction with FITC-conjugated goat anti-rabbit secondary antibody (Life Technologies, Carlsbad, CA, USA, dilution 1:1000). Cells were also stained with VE-Cadherin antibody and then treated with Alexa Fluor 488-conjugated rabbit anti-mouse IgG (Life Technologies, Carlsbad, CA, USA, dilution 1:1000) to exclude endothelial cells contamination. Nuclei were stained with DAPI (Life Technologies, Carlsbad, CA, USA). Stained cells were analysed by immunofluorescent microscope (40× objective).

### Cholesterol loading of rat aortic smooth muscle cells

Cholesterol was delivered to subconfluent RASMCs (passage number ≤5) by cholesterol: methyl-cyclodextrin complexes (Chol:MβCD, C4951; Sigma Aldrich, Saint Louis, MO, USA). Cells were incubated with different concentrations of Chol:MβCD (20, 50 and 100 μg/ml, based on cholesterol weight) in 0.2% BSA for 72 h. After treatment, RASMCs were either lysed for RNA isolation or stained with Oil Red O. For Oil Red staining, RASMCs were fixed with 10% neutral buffer formalin for 30 min, rinsed in 60% isopropanol for 5 min and stained with freshly prepared Oil Red O solution for 20 min. Nuclei were visualized by haematoxylin staining for 30 s. Images were captured with a Nikon Digital Sight DS-2Mv camera coupled to a light inverted microscope (Nikon Instruments Inc., Melville, NY) (20× objective).

### Quantitative RT-PCR analysis of mRNA isolated from plaque material

RNA was isolated by the Rneasy kit (Qiagen, Carlsbad, CA, USA). RNA concentration and purity were determined by NanoDrop 2000 spectrophotometer (Thermo Fisher Scientific, Waltham, MA). Due to the limited amount or low cellularity of plaque material, we obtained sufficient RNA for molecular analysis from 9 out of 18 patients. 80 ng of total RNA were reverse transcribed using the SuperScriptTM III First-Strand Synthesis SuperMix (Life Technologies, Carlsbad, CA, USA) and amplified using the PerfeCTa SYBR Green SuperMix, Rox (Quanta Biosciences, Gaithersburg, MD, USA) with an initial 3 min incubation at 95 °C followed by 40 cycles of amplification: 95 °C for 15 s and 60 °C for 1 min and examined on a 7500 Fast Real-Time PCR system (Applied Biosystems, Life Technologies, Carlsbad, CA, USA). The sequences of primers used are shown in Additional file 1: Table S1. mRNA levels were evaluated by using the ΔCt method. ΔCt values were calculated using ribosomal protein L13 mRNA (RPL13) as an internal reference, and then the highest ΔCt value (the lowest expression level of the target gene observed in patients) was used to calculate ΔΔCt values [45] using the following formula: ΔΔCT_n_ = −(ΔCt_*n*_–ΔCt_*ref*_) +1, where ΔCt_*ref*_ is the ΔCt of the patient with the lowest expression level of the target gene and ΔCt_*n*_ is the ΔCt of patient # *n.*

### Quantitative RT-PCR analysis of microRNA isolated from plaque material

Total RNA was isolated by the MiRneasy FFPE kit (Qiagen, Carlsbad, CA, USA). RNA quantitation was assessed on NanoDrop 2000 (Thermo Fisher Scientific, Waltham, MA, USA). The miScript II RT Kit (Qiagen, Carlsbad, CA, USA) was used to synthesize the cDNA. Then, the miScript SYBR Green PCR Kit (Qiagen, Carlsbad, CA, USA) combined with the miScript Primer Assays (Qiagen, Carlsbad, CA, USA) was used to determine the relative expression levels of the mature miRs of interest: mir21-5p, miR155-5p, miR125a-5p, miR126-5p, miR146-5p, miR424-5p. miR levels were evaluated by using the ΔCt method. ΔCt values were calculated using SNOD61 as an internal reference, and then the highest ΔCt value (the lowest expression level of the target gene observed in patients) was used to calculate ΔΔCt values [45] using the following formula: ΔΔCT_n_ = −(ΔCt_*n*_–ΔCt_*ref*_) +1, where ΔCt_*ref*_ is the ΔCt of the patient with the lowest expression level of the target gene and ΔCt_*n*_ is the ΔCt of patient # *n.*

### Quantitative RT-PCR analysis of mRNA isolated from rat aortic smooth muscle cells

500 ng of total RNA isolated from RASMCs was reverse transcribed and amplified as described above. The sequences of primers used are shown in Additional file 1: Table S1. Relative changes in gene expression were determined by the 2^−ΔΔCt^ formula using RPL13 as reference gene.

### Statistical analysis

Hierarchical clustering was performed using the Genepattern engine (http://genepattern.broadinstitute.org) with pairwise single-linkage as clustering method; column and row distance measures were calculated using Spearman’s rank correlation. The gene and miRNA correlation matrix was generated using the XLSTAT software (http://www.xlstat.com). The miRNA and mRNA levels are expressed as mean ± SEM of at least three independent experiments. Differences between the mean were determined by two-tailed Student’s *t* test and a P < 0.05 was considered to be statistically significant.

## Results

### Characterization of patients

The characteristics of the studied population and of the target lesion are shown in Table 1. Most patients were suffering from hypertension (16/20, 80%) and hypercholesterolemia (17/20, 85%). 14 patients (70%) were diabetic, and 13 patients (65%) were affected by coronary artery disease. The localization of target lesions was heterogeneous: proximal superficial femoral artery (SFA), middle SFA, distal SFA or popliteal 1° segment in 6 (30%), 6 (30%), 4 (20%) and 4 (20%) patients, respectively.

**Table 1:** Patient and lesion characteristics

Variables	Patients (n = 20)
Age mean (SD)	67.05 (8.49)
Male (%)	16 (80)
Diabetes (%)	14 (70)
Hypertension (%)	16 (80)
Hypercolesterolaemia (%)	17 (85)
CAD (%)	13 (65)
Below the knee disease (%)	6 (31.6)
Target lesion	
Proximal SFA (%)	6 (30)
Middle SFA (%)	6 (30)
Distal SFA (%)	4 (20)
Popliteal 1° segment (%)	4 (20)
Total lesion length mean (mm) (SD)	40 (26.2)

*SD* standard deviation, *CAD* coronary artery disease, *SFA* superficial femoral artery

### Characterization of plaque material to evaluate suitability for molecular studies

**Fig. 1:**
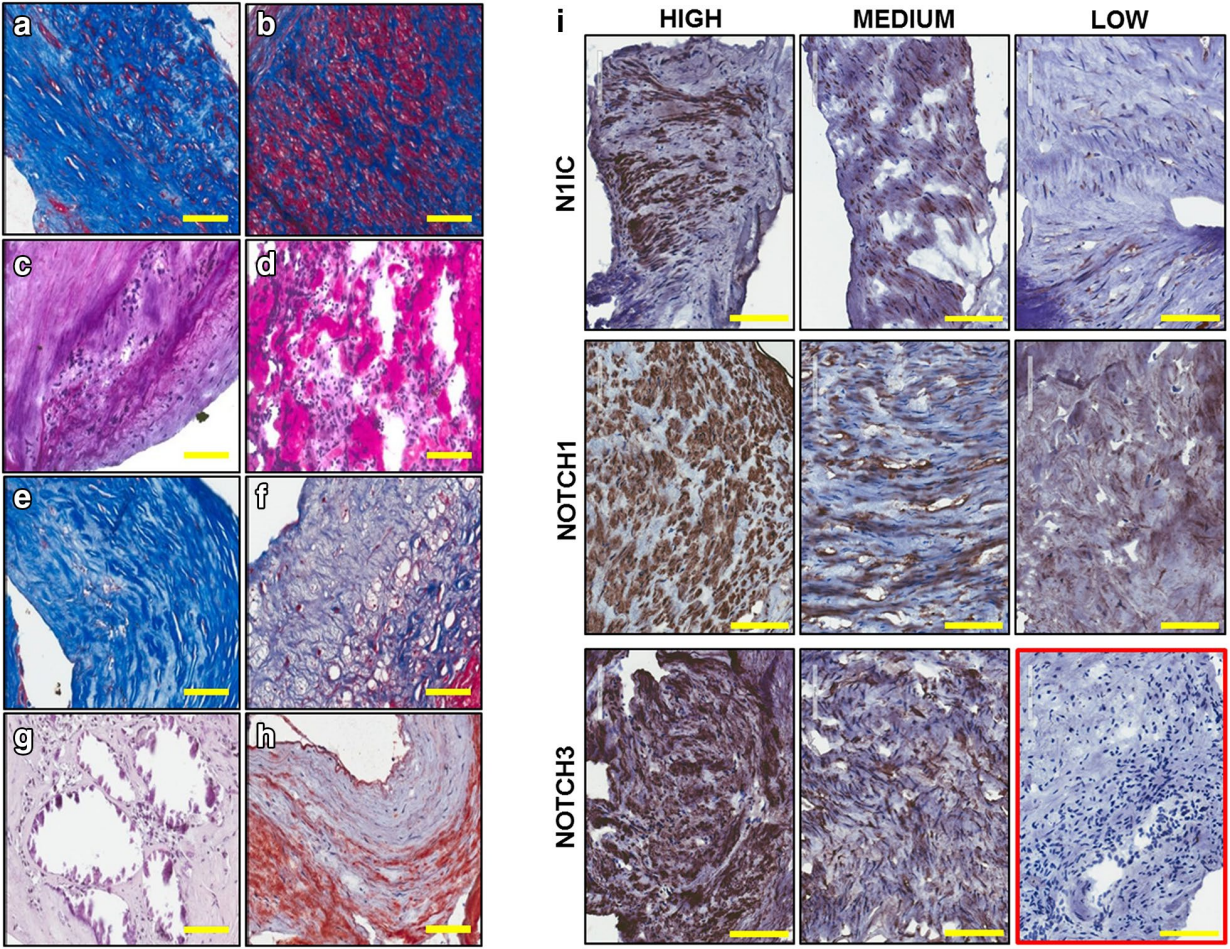
Representative images of histological characteristic of patients’ plaque material. Medium (**a**) and high (**b**) cellularity, medium (**c**) and high (**d**) inflammatory infiltrate, dense relatively acellular fibrous tissue (**e**), loose fibrous tissue (**f**), calcification (**g**) and lipids contents (**h**). Representative images showing different level of expression of Notch receptors (cleaved Notch 1, Notch1, and Notch3) in plaque material. Isotype control is shown in the *red box* (**i**). (Magnification ×4, bar size 100 µm)

Cellularity and inflammatory infiltrate contents were scored based on the estimated percentage of positive cells seen in the whole section [46]. Thus, plaques were graded 1 (less than 10% positive cells, low cellularity), 2 (between 10 and 50%, medium cellularity) and 3 (more than 50% positive cells, high cellularity) (Additional file 1: Table S2). Cellularity of plaque material was low in 3 (15%), medium in 8 (40%) and high in 9 samples (45%). Representative staining of medium and high levels of cellularity are shown in Fig. 1 a and b, respectively. Inflammatory cells, identified by their morphology, were present in variable amounts among patients: low in 11 (55%), medium in 6 (30%) and high in 3 (15%) samples. Medium and high levels of inflammatory cells are shown in Fig. 1c and d, respectively. Using a similar approach, fibrous tissue, calcification, lipids were scored based on the estimated percentage area of tissue section that stained positive. Thus, plaques were graded 1 (less than 5%, low level), 2 (between 5 and 20%, medium level) and 3 (above 20%, high level) (Additional file 1: Table S2). In 1 patient (5%) dense fibrous tissue (Fig. 1e) was present at low level, in other 3 (15%) at medium level and in the remaining 16 (80%) at high level. The majority of samples (13 patients, 65%) showed low levels of loose fibrous tissue (Fig. 1f), whereas in 3 (15%) and in 4 (20%) patients there was medium and high fibrous tissue content, respectively. Only 3 (15%) and 1 (5%) plaques presented medium or high calcification (Fig. 1g), respectively. Lipids content (Fig. 1h) was low in 7 (35%), medium in 10 (50%) and high in 3 samples (15%).

Based on the cellularity content, 18 out of 20 specimens were stained to assess the expression of Notch1 and Notch3 receptors in the plaque material to be used for the RNA studies. For Notch1 and Notch3 detection, two antibodies which recognize the carboxyl termini of the proteins, thus binding to every form of receptor (precursor, transmembrane and intracellular form), were used. We also used an antibody specific for the intracellular, active form of Notch1 (N1IC), cleaved at valine1744. A similar antibody is not available to detect specifically the active form of Notch3. Positivity to antibodies was scored based on the estimated percentage area of tissue section that stained positive. Thus, plaques were graded 0 (no staining), 1 (between 1 and 5%, low level), 2 (between 5 and 20%, medium level) and 3 (above 20%, high level) (Additional file 1: Table S2). Representative images are shown in Fig. 1i. Expression of Notch1 was low in 2 (11.1%), medium in 7 (38.9%) and high in 9 out of 18 tested patients (50%). N1IC staining was medium or high in 3 (16.7%) and 4 (22.2%) patients, respectively. Low or no staining for N1IC was detected in the remaining 11 patients (61.1%). Expression of Notch3 was medium/high in all samples; in particular, in 3 patients (16.7%) was medium and in the remaining 15 patients (83.3%) was high. Positive cells for each Notch receptor tested and for N1IC were constituted by a morphologically heterogenous population including cells with round or spindle-like morphologies.

Based on these analyses, which revealed a high grade of heterogeneity among the samples in positivity for Notch receptors staining, it was determined that plaque material obtained from 18 patients was suitable for RNA studies.

### Analysis of mRNAs and miRs identifies Notch ligand-specific gene expression profile in plaque material

**Fig. 2:**
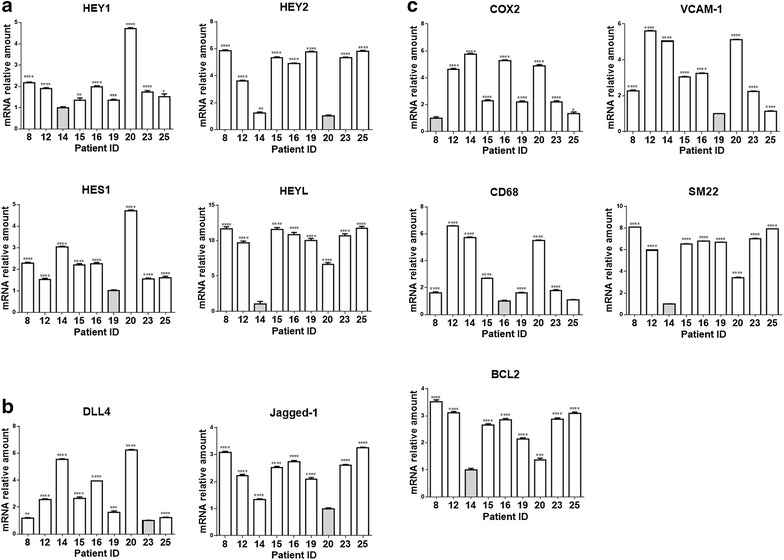
Expression of Notch pathway components and inflammatory markers in plaque material. Relative expression levels of Notch target genes (**a**) and ligands (**b**) and of genes related to vascular smooth muscle cells transdifferentiation (**c**) in plaque material. Relative changes in mRNA expression levels were calculated according to the ΔΔCt method relative to the patient with the lowest expression level of the target gene (*grey bar*) as described in Materials and Methods-Online. ****P < 0.0001, ***P < 0.001, **P < 0.01 and *P < 0.05, significantly different from the control (*grey bar*)

**Fig. 3:**
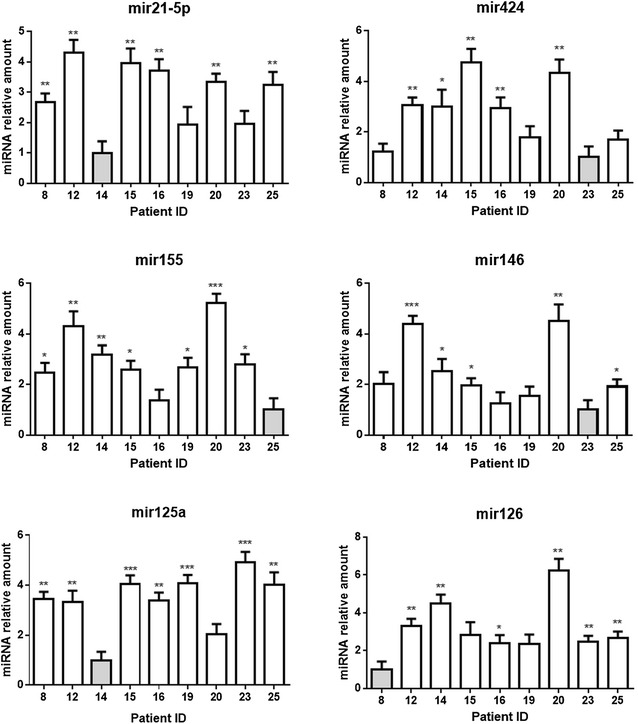
miRs expression levels in plaque material. Relative expression levels of mir21-5p, miR155-5p, miR125a-5p, miR424-5p, miR126-5p and miR146-5p in plaque material. Relative changes in miRNA expression levels were calculated according to the ΔΔCt method relative to the patient with the lowest expression level of the target gene (*grey bar*) as described in Materials and Methods-Online. ***P < 0.001, **P < 0.01 and *P < 0.05, significantly different from the control (*grey bar*)

Nine out of 18 samples gave an RNA yield that allowed molecular analyses. In order to determine the extent and mechanism of Notch receptors activation in plaques, we measured the expression of ligands Dll4 and Jag1 and of target genes Hes1, Hey1, Hey2 and HeyL by quantitative RT-PCR (Fig. 2a, b). We also measured levels of COX2, VCAM1, CD68, SM22, Bcl2 mRNAs (Fig. 2c) and of atherosclerosis/inflammation -related miRs 21-5p, 125a-5p, 126-5p, 146-5p, 155-5p and 424-5p, to compare the inflammation and apoptosis levels among plaque material (Fig. 3).

**Fig. 4:**
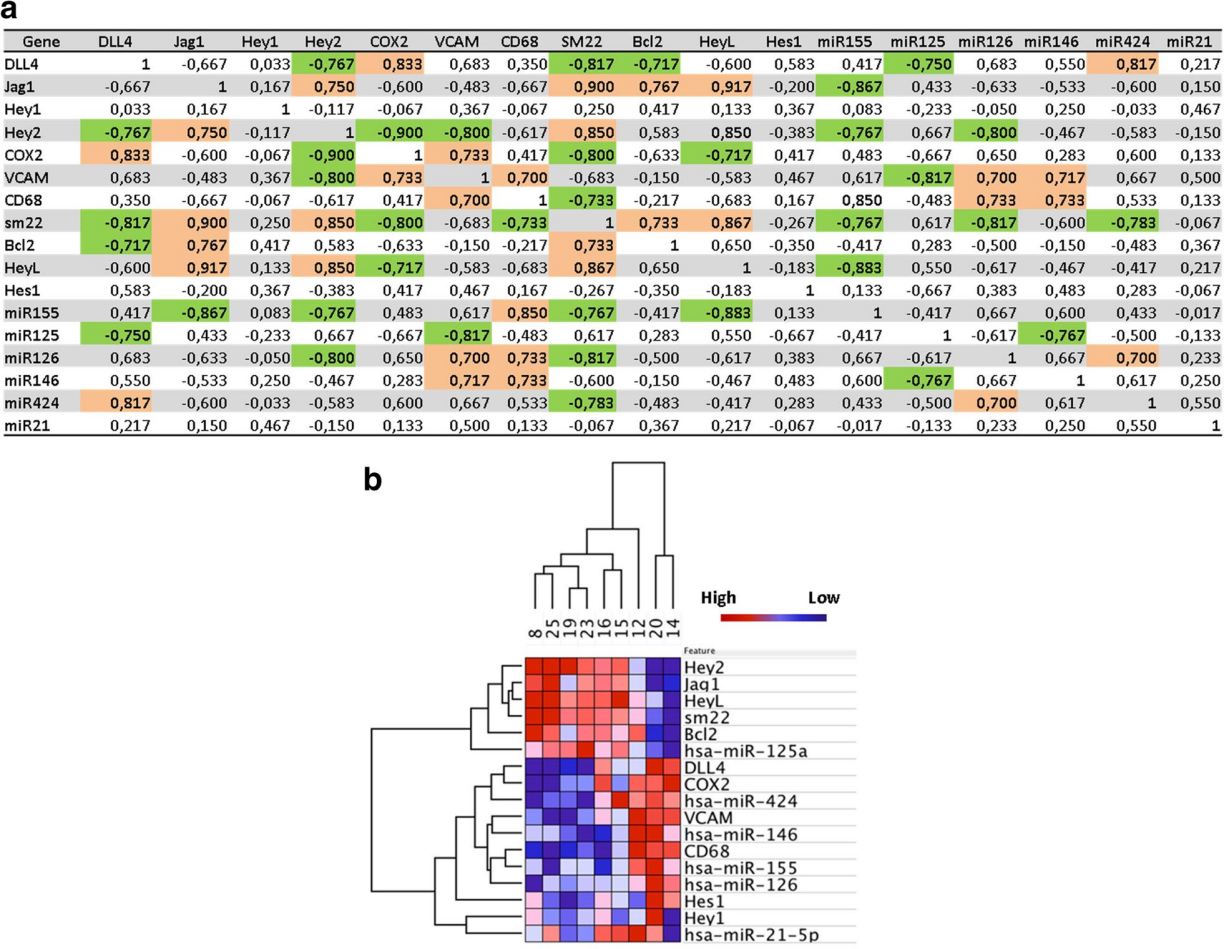
Spearman’s correlation analysis between the expression levels of all the mRNAs and miRNAs tested in plaque material. Spearman’s rank are reported for each correlation. Values in *bold* are different from 0 with a significance level alpha = 0.05. *Orange* and *green boxes* highlight positive and negative correlation, respectively (**a**). Heat map of hierarchical clustering of gene expression in plaque material from each patients. The *columns* indicate patient numbers and the *rows* indicate the analysed genes. Upregulated genes are shown in *red* and downregulated genes are shown in *blue*. The intensity of color is proportional to the relative mRNA and miR levels of transcription (**b**)

The results of correlations analyses among the expression levels of these mRNAs and miRs are reported in Fig. 4a (correlation coefficients) and Additional file 1: Table S3 (associated P values). We found that Dll4 correlated negatively with Hey2 (r = −0.767, P = 0.021) whereas Jag1 correlated positively with Hey2 (r = 0.750, P = 0.025) and HeyL (r = 0.917, P = 0.001). These data suggest that, in our samples, Hey2 and HeyL are downstream of Jag1- but not of Dll4-activated Notch signaling. Dll4 correlated positively with COX2 (r = 0.833; P = 0.008) and miR424-5p (r = 0.817; P = 0.011) (markers of inflammation and plaque instability) and negatively with Bcl2 (r = −0.717; P = 0.037), SM22 (r = −0.817; P = 0.011) and miR125-5p (r = −0.750; P = 0.025) (markers of plaque stability). On the contrary, Jag1 correlated positively with SM22 (r = 0.900; P = 0.002) and negatively with the marker of inflammation miR155-5p (r = −0.867; P = 0.005). These results suggest that Notch activation by Dll4, but not Jag1, is associated to an inflamed and unstable plaque phenotype, whereas Jag1-mediated Notch activation leads to expression of markers of plaque stability.

### Stratification of PAD patients based on plaque gene expression profile

Hierarchical clustering was performed to identify clusters of co-expressed genes and their distribution in the studied patients. As shown in the heat map in Fig. 4b this analysis showed the existence, in the plaque material, of two specific gene expression clusters associated to Notch activation by a specific ligand: an “inflammatory cluster” characterized by high expression of Dll4, CD68, COX2, VCAM1, miR126-5p, miR146-5p, miR155-5p, miR424-5p and a “stability cluster” characterized by high expression of Jag1, Hey2, HeyL, SM22, Bcl2 and miR125a-5p. Patients characterized by high level of Dll4, CD68, VCAM1, COX2 miR126-5p, miR146-5p, miR155-5p and miR424-5p (“inflammatory” profile) showed low levels of Jag1, Hey2, HeyL, SM22, Bcl2 and miR125a-5p (patients 14 and 20), while other patients (patients 8, 23 and 25) showed an opposite pattern (“stability” profile) characterized by low levels of Dll4, CD68, COX2, VCAM1, miR126-5p, miR146-5p, miR155-5p, miR424-5p and high levels of Jag1, Hey2, HeyL, SM22, Bcl2, and miR125a-5p. Patients 12, 15, 16 and 19 presented mixed/intermediate gene expression values.

**Fig. 5:**
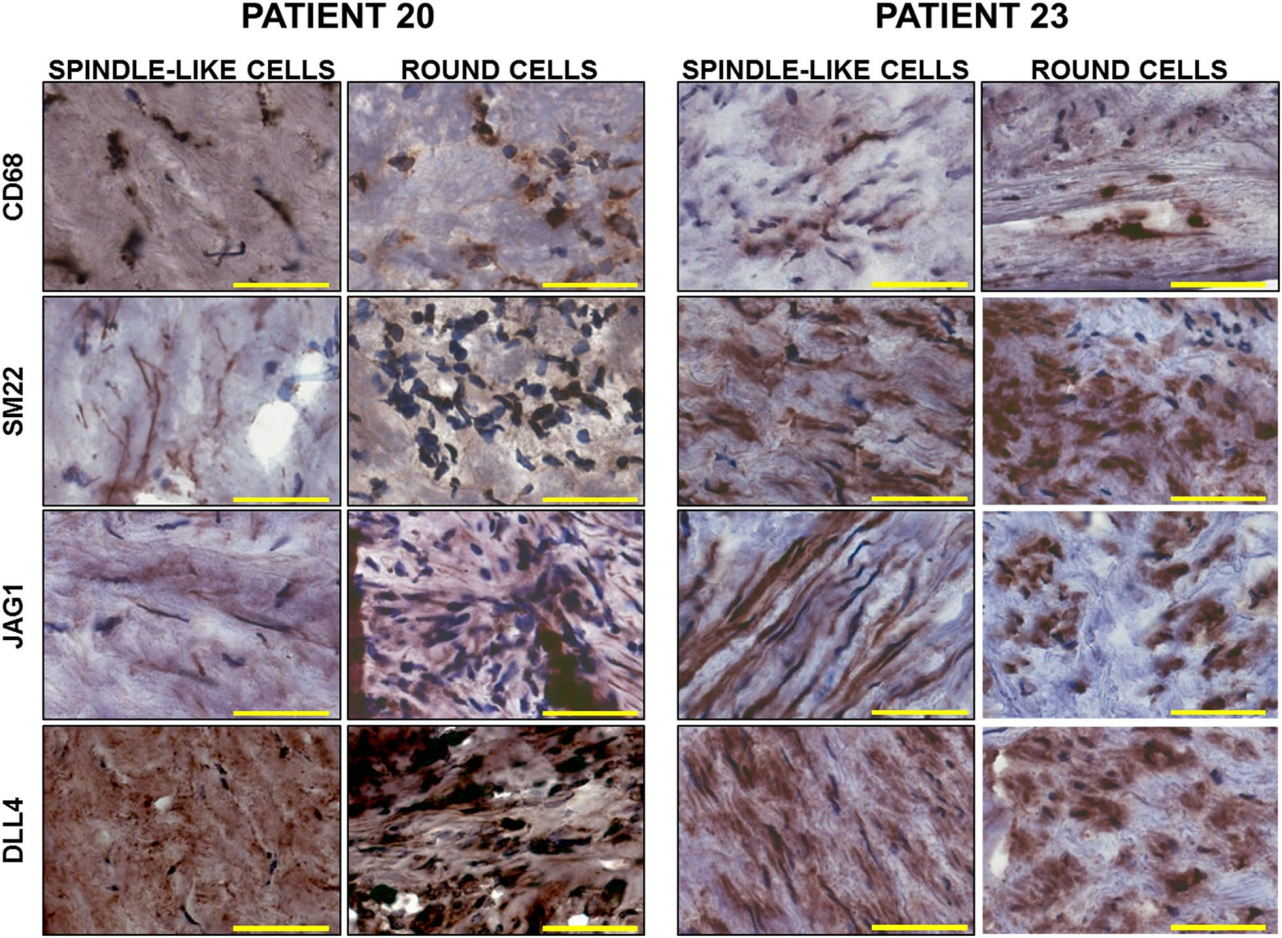
Immunostaining for CD68, αSMA, Jag1 and Dll4 in patients with stable or inflammatory plaque profile. Representative images comparing expression of CD68, αSMA, Jag1 and Dll4 in different cell types (spindle-like or round cells) in plaque material from patient 20 (stable plaque) and patient 23 (inflamed) (magnification ×20, bar size 50 µm)

To determine whether the predominance of macrophages or VSMCs contributed to the intra-plaque Jag1- and Dll4-mediated Notch signaling signatures, we performed immunostaining for Jag1, Dll4, SM22 and CD68 on frozen sections of plaque material from patients 20 and 23, representative of the “inflamed” and “stable” profiles, respectively. In agreement with the RNA studies, immunostaining showed higher expression of CD68 and Dll4, together with a lower expression of SM22 and Jag1 in patient 20 compared to patient 23 (Fig. 5). In both patients, CD68 and Dll4 staining was present in round, macrophage-like cells, as well as in spindle-like cells (Fig. 5). Similarly, we detected both round and elongated spindle-like cells positive for SM22 and Jag1 (Fig. 5). These experiments suggest that the observed gene expression profile was determined by a heterogeneous cells population expressing different levels of the genes of interest rather than by the prevalence of macrophages or VSMCs in the analyzed material.

Our in vivo findings were suggestive of a trans-differentiation of VSMCs into macrophage-like cells, which could, at least partially, be mediated by a switch from a Jag1- to Dll4-Notch activated signaling. To test this hypothesis we induced trans-differentiation of VSMCs to macrophage-like cells by cholesterol loading as described [47].

### Effects of cholesterol loading on Notch signaling in rat aortic smooth muscle cells

**Fig. 6:**
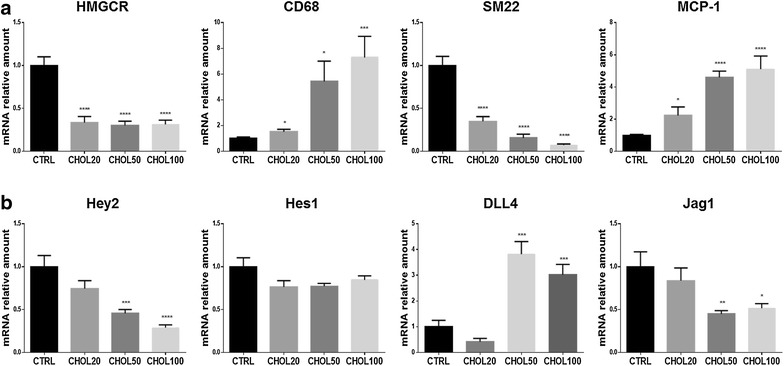
mRNAs expression levels affected by cholesterol loading in rat aortic smooth muscle cells. Relative expression levels of HMGCR, CD68, SM22, MCP-1 (**a**), Hey2, Hes1, Dll4 and Jag1 (**b**) in RASMCs treated with 20 (CHOL20), 50 (CHOL50) and 100 (CHOL100) μg/ml of cholesterol for 72 h. Relative changes in mRNA expression levels were calculated according to the ΔΔCt method. Results are expressed as mean ± SEM of three independent experiments, each performed in triplicate. ****P < 0.0001, ***P < 0.001, **P < 0.01 and *P < 0.05, significantly different from the control (CTRL)

We determined the expression levels of Jag1, Dll4, Hes1, Hey2, HeyL in rat aortic smooth muscle cells (RASMCs) loaded with cholesterol. We first checked the purity of our RASMCs culture by performing α-SM-actin and VE-Cadherin stainings (Additional file 1: Figure S1) and confirmed the presence of lipid droplets throughout the cytosol of most cells incubated with increasing amounts of cholesterol: methyl-cyclodextrin complexes (Chol: MβCD) (Additional file 1: Figure S2). As expected, we found down-regulation of HMG-CoA reductase (the enzyme involved in the synthesis of cholesterol) and SM22 mRNAs (0.33- and 0.35-fold, respectively) and up-regulation of CD68 and MCP1 (monocyte chemotactic protein 1) (1.51- and 2.22-fold, respectively) in cells exposed to 20 µg/ml of Chol:MβCD in comparison with untreated cells. Differences in the expression of these genes were maintained or increased in cells treated with higher amounts of cholesterol (HMG-CoA reductase: 0.30- and 0.31-fold, CD68: 5.44- and 7.29-fold, MCP1: 4.6- and 5.11-fold and SM22: 0.15- and 0.07-fold, in the presence of 50 and 100 µg/ml of Chol:MβCD, respectively) (Fig. 6a). Additionally, we found down-regulation of Hey2 (0.45- and 0.29-fold) and Jag-1 (0.45- and 0.51-fold) and up-regulation of Dll4 (3.82- and 3.02-fold) in RASMCs treated with 50 and 100 µg/ml of Chol:MβCD, respectively, in comparison with untreated cells (Fig. 6b). These data show that the transdifferentiation of RASMCs in macrophage-like cell induced by cholesterol loading is paralleled by a shift from a Jag1- to a Dll4-mediated Notch signaling.

### Clinical Follow-up

Three patients were lost to 6 months follow-up (patients 11, 14 and 18) and one patient was lost to 1 year follow-up (patient 12). At 6 months 10 patients were asymptomatic with no ischemic events (patients 2, 4, 5, 8, 13, 15,17,19, 22 and 25), 3 suffered from recurrence of claudication at the treated leg (patients 12, 16 and 22) and 3 from new symptoms at the other leg (patients 4, 12 and 20). In 3 patients a coronary event occurred (patients 10, 21 and 23). In one patient, an aneurism occurred at the site of atherectomy, which was corrected by a targeted procedure (patient 24) (Additional file 1: Table S4). At 1 year, symptoms recurred at the same leg in 3 patients (patient 16, 20 and 22), 2 patients had a claudication started at the contralateral leg (patients 2 and 4) and in 3 patients a coronary event occurred (patients 10, 13 and 20) (Additional file 1: Table S4). No patients died.

## Discussion

In this study, we have found that PAD patients could be stratified into three groups based on the type on Notch signaling present in plaque material: a group characterized by high Jag1/Hey2/HeyL and low Dll4 (high-Jag1), a group characterized by high Dll4, low Jag1/Hey2/HeyL (high-Dll4) and a group with intermediate characteristics between the first two. These three groups of patients showed specific gene expressions profiles. A “stable” plaque profile, characterized by high SM22, Bcl2, miR125a-5p, was associated to high-Jag1, an “inflammatory” plaque profile, characterized by high CD68, VCAM1, COX2, miR155-5p, 126-5p, 146-5p, 424-5p, associated to high-Dll4, and a “mixed” plaque profile in the group with intermediate values. These observations reveal a possible role of Notch signaling in the pathophysiology of PAD and suggest that ligand-specific activation of this pathway could determine the plaque characteristics and therefore progression of the disease.

Inflammatory cytokines-induced Dll4/Notch3 signaling leads to macrophages activation [28] whereas Jag1/Notch1, 3 signaling promotes maturation of VSMCs [18–21]. SM22 is a direct transcriptional target of Notch in VSMCs [18] but Hey2 has also been involved in the modulation of the differentiation of VSMCs [21]. Activation of Notch by Jag1 counteracts IL-1β-induced transdifferentiation of VSMCs from a quiescent/contractile to a proliferative/secretory phenotype [24] by upregulation of Hey1 and HeyL [23]. On the contrary, Dll4-mediated Notch activation is not able to induce differentiation of mesenchymal cells into VSMCs [48]. Taking these studies into consideration, the existence of the different Notch signaling profiles in our samples might be explained by a prevalence of macrophages in the high-Dll4 group and of VSMCs in the high-Jag1. Consistent with this hypothesis, we found a positive correlation between Dll4 and CD68 and between Jag1 and SM22 mRNA levels. However, immunohistochemical analysis on plaque material from one high-Dll4 patient and one high-Jag1 patient showed a heterogeneous cells population consisting of spindle-like and round cells, both types expressing variable amount of Dll4, CD68, Jag1, SM22, Notch1 and Notch3. This is consistent with capturing VSMCs at different stages of transdifferentiation into macrophage-like cells. It is becoming widely recognized that intraplaque cells identified as macrophages can derive from VSMCs acquiring macrophage-like morphology and phagocytic activity [49–53]. VSMCs expressing variable amounts of both SM22 and CD68 have been identified in aortic [51] and coronary [50] atherosclerotic plaques. From our results, we hypothesize that Dll4 induction in VSMCs and the switch from a Jag1-activated to a Dll4-activated Notch signaling could be a marker or a causative factor of VMSCs transdifferentiation in PAD plaques. We found that COX2 and VCAM1 correlated positively with Dll4 and negatively with Jag1. Jag1/Notch inhibition has been linked to COX2 induction in VSMCs [24] and Dll4-mediated Notch signaling in macrophages activates NF-k B [28, 54], which, in turn, induces VCAM1 and COX2 transcription (http://www.bu.edu/nf-kb/gene-resources/target-genes/). We also found that Bcl2 correlated positively with Jag1 and negatively with Dll4. It has been shown that Notch activation in VSMCs is involved in transcriptional induction of the Bcl2-family of protein [55] and survival [22] but there is no evidence linking a specific Notch ligand to the pro-survival activity of this pathway.

Consistent with a role for Notch in VSMCs trans-differentiation into macrophages-like cells, we found that cholesterol loading of RASMCs led to reduction of contractility and induction of inflammatory markers in association with reduced levels of Jag1 and Hey2 and increased levels of Dll4 mRNAs. Further studies should determine the molecular mechanisms underlying the cholesterol-induced Jag1–Dll4 switch and whether this switch of ligands, alone or in synergism with other factors (i.e. endothelium secreted-miR126, circulating glucose levels, hemodynamics), plays a role in the transdifferentiation of VSMCs into macrophages-like cells.

We detected high levels of pro-inflammatory miR126-5p, 146-5p, 155-5p and 424-5p and low level of anti-inflammatory miR125a-5p in high-Dll4 patients. In addition, our analysis showed a negative correlation between Jag1 and miR155-5p suggesting that Jag1, but not Dll4, could be the ligand involved in the Notch-mediated downregulation of miR155-5p [41]. We found that Dll4 positively correlated with miR424-5p, consistent with a study showing that hypoxic conditions, also present in plaques, lead to increased miR424-5p which stabilizes hypoxia-inducible factor-2 α (HIF-2α) [56], required for Dll4 expression [57]. In our study, Dll4 was also negatively correlated with miR125a: interestingly, miR125a downregulates Lunatic Fringe, a glycosylation enzyme which determines the selective response of Notch receptors to different ligands [58].

Our study suggests the existence of a Jag1-activated Notch signaling, associated to quiescence/contractility of VSMCs intra-plaque and low levels of inflammation, and a Dll4-activated Notch signaling associated to markers of inflammation, which could be involved in the progression of the disease. Consistently, Delta-like ligands have been shown to activate small mother against decapentaplegic (SMAD) signaling [59] which has been linked to restenosis of superficial femoral artery [60]. The existence of an “inflamed” or “stable” plaque signature has been reported by a microarray-based study of 101 PAD specimens and the Notch target gene Hey2 was included in the “stable” signature [61]. Microarray analysis on laser-microdissected samples of atheroma containing a prevalence of macrophages or VSMCs led the authors to link the “inflamed” phenotype to the predominance of macrophagic component and “stable” phenotype to a prevalence of VSMCs [61]. There are several other examples of specific phenotypes associated to a specific combination of Notch ligands and receptors. Jag1 and Dll4 have opposite effects on sprouting angiogenesis [13] and differential effects of Jag1 and Dll1,4 have been described on T cells proliferation [15] and the regulation of T cell effector functions in autoimmunity [14]. Furthermore, there is evidence of differential regulation of osteoclastogenesis by Notch2/Dll1 and Notch1/Jagged1 axes [16].

Clinical follow-up showed that patients with a “stable” plaque profile (Jag1-activated Notch) were all asymptomatic at 6- and 12-months, whereas patients with an “inflamed” (Dll4-activated Notch) or “mixed” (with high Dll4/COX2 or high COX2) profile presented symptoms to the treated or to the other leg (Additional file 1: Table S4). If confirmed in a larger set of patients, our findings, linking a specific Notch signaling to plaque phenotype, could have clinical relevance. To this end it is important to mention that soluble Dll4-mediated Notch signaling blockade interferes with atherosclerosis progression in an animal model of metabolic syndrome [54] and that antibodies antagonizing Dll4-Notch signaling, are already under clinical investigation in the oncology setting [62]. We also found that the expression of several intra-plaque miRNAs is associated to Jag1 or Dll4 levels. Circulating miRNAs are being investigated as possible biomarkers for early detection and progression of PAD [63]. Since miR126-5p is secreted by endothelial cells [31], the high levels of miRNA126-5p observed in the “inflammatory” plaques could be mirrored in the serum levels of this miRNA. Further studies are needed to investigate this possibility and to assess the clinical utility of the circulating level of miR126-5p.

## Conclusions

The Notch pathway plays a role in atherosclerosis but its involvement in the progression of PAD has not been investigated. We report that plaque material from PAD patients is characterized by “stable plaque “ or “inflamed plaque” gene expression profiles associated to Notch activation by ligand Jagged 1 or Delta like 4, respectively. Clinical follow up suggests that Delta like 4 ligand-associated signature could have unfavourable effects on the progression of the disease. Our data, if confirmed in a larger study, could be translationally relevant since pharmacological Dll4 inhibitors have been developed and are entering clinical trials for solid tumors.

## Additional file

**Additional file 1: Figure S1.** Characterization of primary cultures of rat aortic smooth muscle cells by immunofluorescent staining. **Figure S2.** Oil-red staining of cholesterol loaded rat aortic smooth muscle cells. **Table S1.** Primers used for quantitative RT-PCR. **Table S2.** Semiquantitative evaluation of histological characteristics and Notch receptors immunostainings. **Table S3**. P values associated to the results of correlations analyses among the expression levels of mRNAs and miRs analyzed. **Table S4.** Clinical follow up and molecular analysis.

## Abbreviations

Bcl2B: cell lymphoma gene 2
Chol MβCD: cholesterol:methyl-cyclodextrin
COX2: cyclooxygenase 2
CSL: CBF-1, Suppressor of Hairless and Lag-1
DAPI: 4′,6-diamidino-2-phenylindole
Dll4: Delta-like ligand 4
FFPE: formalin fixed paraffin embedded
FITC: fluorescein isothiocyanate
Hes: hairy/enhancer of split
Hey: Hes-related proteins
HIF-2: hypoxia-inducible factor-2
HMG-CoA reductase: 3-hydroxy-3-methyl-glutaryl-co-enzyme A reductase
IL 1-β: interleukin 1-β
Jag1: Jagged1
MCP1: monocyte chemotactic protein 1
NF-kappa B: nuclear factor kappa-light-chain-enhancer of activated B cells
oxLDL: oxidized low density lipoproteins
PAD: peripheral artery disease,
qRT-PCR: quantitative reverse transcriptase-polymerase chain reaction
RASMCs: Rat aortic smooth muscle cells
SM22: smooth muscle-specific protein 22
SMAD: small mother against decapentaplegic
VCAM1: vascular cell adhesion molecule 1
VSMCs: vascular smooth muscle cells

## References

[1] Fowkes FG, Rudan D, Rudan I, Aboyans V, Denenberg JO, McDermott MM, Norman PE, Sampson UK, Williams LJ, Mensah GA, Criqui MH (2013). Comparison of global estimates of prevalence and risk factors for peripheral artery disease in 2000 and 2010: a systematic review and analysis. Lancet.

[2] Armstrong EJ, Sab S, Singh GD, Lim W, Yeo KK, Waldo SW, Patel M, Reeves R, MacGregor JS, Low RI, Shunk KA, Mahmud E, Rogers JH (2014). Predictors and outcomes of recurrent stent thrombosis: results from a multicenter registry. JACC Cardiovasc Interv.

[3] Gur I, Lee W, Akopian G, Rowe VL, Weaver FA, Katz SG (2011). Clinical outcomes and implications of failed infrainguinal endovascular stents. J Vasc Surg.

[4] Schillinger M, Minar E (2005). Restenosis after percutaneous angioplasty: the role of vascular inflammation. Vasc Health Risk Manag.

[5] Lusis S (2000). Update on restraint use in acute care settings. Plast Surg Nurs.

[6] Clarke M, Bennett M (2006). The emerging role of vascular smooth muscle cell apoptosis in atherosclerosis and plaque stability. Am J Nephrol.

[7] Inoue T, Node K (2009). Molecular basis of restenosis and novel issues of drug-eluting stents. Circ J.

[8] Boucher J, Gridley T, Liaw L (2012). Molecular pathways of notch signaling in vascular smooth muscle cells. Front Physiol.

[9] Rizzo P: The Notch pathway: A new therapeutic target in atherosclerosis?; in Ferrari R, (ed): 2015, p A74-A76.

[10] Espinoza I, Miele L (2013). Notch inhibitors for cancer treatment. Pharmacol Ther.

[11] Rizzo P, Osipo C, Foreman K, Golde T, Osborne B, Miele L (2008). Rational targeting of Notch signaling in cancer. Oncogene.

[12] Li K, Li Y, Wu W, Gordon WR, Chang DW, Lu M, Scoggin S, Fu T, Vien L, Histen G, Zheng J, Martin-Hollister R, Duensing T, Singh S, Blacklow SC, Yao Z, Aster JC, Zhou BB (2008). Modulation of Notch signaling by antibodies specific for the extracellular negative regulatory region of NOTCH3. J Biol Chem.

[13] Benedito R, Roca C, Sorensen I, Adams S, Gossler A, Fruttiger M, Adams RH (2009). The notch ligands Dll4 and Jagged1 have opposing effects on angiogenesis. Cell.

[14] Elyaman W, Bradshaw EM, Wang Y, Oukka M, Kivisakk P, Chiba S, Yagita H, Khoury SJ (2007). JAGGED1 and delta1 differentially regulate the outcome of experimental autoimmune encephalomyelitis. J Immunol.

[15] Rutz S, Mordmuller B, Sakano S, Scheffold A (2005). Notch ligands Delta-like1, Delta-like4 and Jagged1 differentially regulate activation of peripheral T helper cells. Eur J Immunol.

[16] Sekine C, Koyanagi A, Koyama N, Hozumi K, Chiba S, Yagita H (2012). Differential regulation of osteoclastogenesis by Notch2/Delta-like 1 and Notch1/Jagged1 axes. Arthritis Res Ther.

[17] van de Walle I, Waegemans E, De MJ, De SG, De SM, Snauwaert S, Vandekerckhove B, Kerre T, Leclercq G, Plum J, Gridley T, Wang T, Koch U, Radtke F, Taghon T (2013). Specific Notch receptor-ligand interactions control human TCR-alphabeta/gammadelta development by inducing differential Notch signal strength. J Exp Med.

[18] Boucher JM, Peterson SM, Urs S, Zhang C, Liaw L (2011). The miR-143/145 cluster is a novel transcriptional target of Jagged-1/Notch signaling in vascular smooth muscle cells. J Biol Chem.

[19] Domenga V, Fardoux P, Lacombe P, Monet M, Maciazek J, Krebs LT, Klonjkowski B, Berrou E, Mericskay M, Li Z, Tournier-Lasserve E, Gridley T, Joutel A (2004). Notch3 is required for arterial identity and maturation of vascular smooth muscle cells. Genes Dev.

[20] Liu H, Kennard S, Lilly B (2009). NOTCH3 expression is induced in mural cells through an autoregulatory loop that requires endothelial-expressed JAGGED1. Circ Res.

[21] Tang Y, Urs S, Liaw L (2008). Hairy-related transcription factors inhibit Notch-induced smooth muscle alpha-actin expression by interfering with Notch intracellular domain/CBF-1 complex interaction with the CBF-1-binding site. Circ Res.

[22] Sweeney C, Morrow D, Birney YA, Coyle S, Hennessy C, Scheller A, Cummins PM, Walls D, Redmond EM, Cahill PA (2004). Notch 1 and 3 receptor signaling modulates vascular smooth muscle cell growth, apoptosis, and migration via a CBF-1/RBP-Jk dependent pathway. FASEB J.

[23] Keuylian Z, de Baaij JH, Gueguen M, Glorian M, Rouxel C, Merlet E, Lipskaia L, Blaise R, Mateo V, Limon I (2012). The Notch pathway attenuates interleukin 1beta (IL1beta)-mediated induction of adenylyl cyclase 8 (AC8) expression during vascular smooth muscle cell (VSMC) trans-differentiation. J Biol Chem.

[24] Clement N, Gueguen M, Glorian M, Blaise R, Andreani M, Brou C, Bausero P, Limon I (2007). Notch3 and IL-1beta exert opposing effects on a vascular smooth muscle cell inflammatory pathway in which NF-kappaB drives crosstalk. J Cell Sci.

[25] Monsalve E, Perez MA, Rubio A, Ruiz-Hidalgo MJ, Baladron V, Garcia-Ramirez JJ, Gomez JC, Laborda J, Diaz-Guerra MJ (2006). Notch-1 up-regulation and signaling following macrophage activation modulates gene expression patterns known to affect antigen-presenting capacity and cytotoxic activity. J Immunol.

[26] Palaga T, Buranaruk C, Rengpipat S, Fauq AH, Golde TE, Kaufmann SH, Osborne BA (2008). Notch signaling is activated by TLR stimulation and regulates macrophage functions. Eur J Immunol.

[27] Xu J, Chi F, Guo T, Punj V, Lee WN, French SW, Tsukamoto H (2015). NOTCH reprograms mitochondrial metabolism for proinflammatory macrophage activation. J Clin Invest.

[28] Fung E, Tang SM, Canner JP, Morishige K, Arboleda-Velasquez JF, Cardoso AA, Carlesso N, Aster JC, Aikawa M (2007). Delta-like 4 induces notch signaling in macrophages: implications for inflammation. Circulation.

[29] Nakano T, Fukuda D, Koga JI, Aikawa M (2016). Delta-like ligand 4-Notch signaling in macrophage activation. Arterioscler Thromb Vasc Biol.

[30] Chen LJ, Lim SH, Yeh YT, Lien SC, Chiu JJ (2012). Roles of microRNAs in atherosclerosis and restenosis. J Biomed Sci.

[31] Zhou J, Li YS, Nguyen P, Wang KC, Weiss A, Kuo YC, Chiu JJ, Shyy JY, Chien S (2013). Regulation of vascular smooth muscle cell turnover by endothelial cell-secreted microRNA-126: role of shear stress. Circ Res.

[32] Merlet E, Atassi F, Motiani RK, Mougenot N, Jacquet A, Nadaud S, Capiod T, Trebak M, Lompre AM, Marchand A (2013). miR-424/322 regulates vascular smooth muscle cell phenotype and neointimal formation in the rat. Cardiovasc Res.

[33] Chen YC, Bui AV, Diesch J, Manasseh R, Hausding C, Rivera J, Haviv I, Agrotis A, Htun NM, Jowett J, Hagemeyer CE, Hannan RD, Bobik A, Peter K (2013). A novel mouse model of atherosclerotic plaque instability for drug testing and mechanistic/therapeutic discoveries using gene and microRNA expression profiling. Circ Res.

[34] Cao J, Zhang K, Zheng J, Dong R (2015). MicroRNA-146a and -21 cooperate to regulate vascular smooth muscle cell proliferation via modulation of the Notch signaling pathway. Mol Med Rep.

[35] Fan X, Wang E, Wang X, Cong X, Chen X (2014). MicroRNA-21 is a unique signature associated with coronary plaque instability in humans by regulating matrix metalloproteinase-9 via reversion-inducing cysteine-rich protein with Kazal motifs. Exp Mol Pathol.

[36] Raitoharju E, Lyytikainen LP, Levula M, Oksala N, Mennander A, Tarkka M, Klopp N, Illig T, Kahonen M, Karhunen PJ, Laaksonen R, Lehtimaki T (2011). miR-21, miR-210, miR-34a, and miR-146a/b are up-regulated in human atherosclerotic plaques in the Tampere Vascular Study. Atherosclerosis.

[37] Nazari-Jahantigh M, Egea V, Schober A, Weber C (2014). MicroRNA-specific regulatory mechanisms in atherosclerosis. J Mol Cell Cardiol.

[38] Chen T, Huang Z, Wang L, Wang Y, Wu F, Meng S, Wang C (2009). MicroRNA-125a-5p partly regulates the inflammatory response, lipid uptake, and ORP9 expression in oxLDL-stimulated monocyte/macrophages. Cardiovasc Res.

[39] Huang F, Zhu X, Hu XQ, Fang ZF, Tang L, Lu XL, Zhou SH (2013). Mesenchymal stem cells modified with miR-126 release angiogenic factors and activate Notch ligand Delta-like-4, enhancing ischemic angiogenesis and cell survival. Int J Mol Med.

[40] Schober A, Nazari-Jahantigh M, Wei Y, Bidzhekov K, Gremse F, Grommes J, Megens RT, Heyll K, Noels H, Hristov M, Wang S, Kiessling F, Olson EN, Weber C (2014). MicroRNA-126-5p promotes endothelial proliferation and limits atherosclerosis by suppressing Dlk1 3. Nat Med.

[41] Wang L, Zhang H, Rodriguez S, Cao L, Parish J, Mumaw C, Zollman A, Kamoka MM, Mu J, Chen DZ, Srour EF, Chitteti BR, HogenEsch H, Tu X, Bellido TM, Boswell HS, Manshouri T, Verstovsek S, Yoder MC, Kapur R, Cardoso AA, Carlesso N (2014). Notch-dependent repression of miR-155 in the bone marrow niche regulates hematopoiesis in an NF-kappaB-dependent manner 2. Cell Stem Cell.

[42] Verhoeven BA, Velema E, Schoneveld AH, Vries JP, de Bruin P, Seldenrijk CA, de Kleijn DP, Busser E, van der Graaf YV, Mol F, Pasterkamp G (2004). Athero-express: differential atherosclerotic plaque expression of mRNA and protein in relation to cardiovascular events and patient characteristics. Rationale and design. Eur J Epidemiol.

[43] Kragel AH, Reddy SG, Wittes JT, Roberts WC (1989). Morphometric analysis of the composition of atherosclerotic plaques in the four major epicardial coronary arteries in acute myocardial infarction and in sudden coronary death. Circulation.

[44] Xu S, Fu J, Chen J, Xiao P, Lan T, Le K, Cheng F, He L, Shen X, Huang H, Liu P (2009). Development of an optimized protocol for primary culture of smooth muscle cells from rat thoracic aortas. Cytotechnology.

[45] Tilley AE, Harvey BG, Heguy A, Hackett NR, Wang R, O’Connor TP, Crystal RG (2009). Down-regulation of the notch pathway in human airway epithelium in association with smoking and chronic obstructive pulmonary disease. Am J Respir Crit Care Med.

[46] Alpizar-Alpizar W, Nielsen BS, Sierra R, Illemann M, Ramirez JA, Arias A, Duran S, Skarstein A, Ovrebo K, Lund LR, Laerum OD (2010). Urokinase plasminogen activator receptor is expressed in invasive cells in gastric carcinomas from high- and low-risk countries. Int J Cancer.

[47] Rong JX, Shapiro M, Trogan E, Fisher EA (2003). Transdifferentiation of mouse aortic smooth muscle cells to a macrophage-like state after cholesterol loading. Proc Natl Acad Sci USA.

[48] Doi H, Iso T, Sato H, Yamazaki M, Matsui H, Tanaka T, Manabe I, Arai M, Nagai R, Kurabayashi M (2006). Jagged1-selective notch signaling induces smooth muscle differentiation via a RBP-Jkappa-dependent pathway. J Biol Chem.

[49] Albarran-Juarez J, Kaur H, Grimm M, Offermanns S, Wettschureck N (2016). Lineage tracing of cells involved in atherosclerosis. Atherosclerosis.

[50] Allahverdian S, Chehroudi AC, McManus BM, Abraham T, Francis GA (2014). Contribution of intimal smooth muscle cells to cholesterol accumulation and macrophage-like cells in human atherosclerosis. Circulation.

[51] Andreeva ER, Pugach IM, Orekhov AN (1997). Subendothelial smooth muscle cells of human aorta express macrophage antigen in situ and in vitro. Atherosclerosis.

[52] Feil S, Fehrenbacher B, Lukowski R, Essmann F, Schulze-Osthoff K, Schaller M, Feil R (2014). Transdifferentiation of vascular smooth muscle cells to macrophage-like cells during atherogenesis. Circ Res.

[53] Vengrenyuk Y, Nishi H, Long X, Ouimet M, Savji N, Martinez FO, Cassella CP, Moore KJ, Ramsey SA, Miano JM, Fisher EA (2015). Cholesterol loading reprograms the microRNA-143/145-myocardin axis to convert aortic smooth muscle cells to a dysfunctional macrophage-like phenotype. Arterioscler Thromb Vasc Biol.

[54] Fukuda D, Aikawa E, Swirski FK, Novobrantseva TI, Kotelianski V, Gorgun CZ, Chudnovskiy A, Yamazaki H, Croce K, Weissleder R, Aster JC, Hotamisligil GS, Yagita H, Aikawa M (2012). Notch ligand delta-like 4 blockade attenuates atherosclerosis and metabolic disorders. Proc Natl Acad Sci USA.

[55] Morrow D, Cullen JP, Cahill PA, Redmond EM (2007). Cyclic strain regulates the Notch/CBF-1 signaling pathway in endothelial cells: role in angiogenic activity. Arterioscler Thromb Vasc Biol.

[56] Ghosh G, Subramanian IV, Adhikari N, Zhang X, Joshi HP, Basi D, Chandrashekhar YS, Hall JL, Roy S, Zeng Y, Ramakrishnan S (2010). Hypoxia-induced microRNA-424 expression in human endothelial cells regulates HIF-alpha isoforms and promotes angiogenesis. J Clin Invest.

[57] Skuli N, Liu L, Runge A, Wang T, Yuan L, Patel S, Iruela-Arispe L, Simon MC, Keith B (2009). Endothelial deletion of hypoxia-inducible factor-2alpha (HIF-2alpha) alters vascular function and tumor angiogenesis. Blood.

[58] Riley MF, Bochter MS, Wahi K, Nuovo GJ, Cole SE (2013). Mir-125a-5p-mediated regulation of Lfng is essential for the avian segmentation clock. Dev Cell.

[59] Hiratochi M, Nagase H, Kuramochi Y, Koh CS, Ohkawara T, Nakayama K (2007). The Delta intracellular domain mediates TGF-beta/Activin signaling through binding to Smads and has an important bi-directional function in the Notch-Delta signaling pathway. Nucleic Acids Res.

[60] Edlin RS, Tsai S, Yamanouchi D, Wang C, Liu B, Kent KC (2009). Characterization of primary and restenotic atherosclerotic plaque from the superficial femoral artery: potential role of Smad3 in regulation of SMC proliferation. J Vasc Surg.

[61] Puig O, Yuan J, Stepaniants S, Zieba R, Zycband E, Morris M, Coulter S, Yu X, Menke J, Woods J, Chen F, Ramey DR, He X, O’Neill EA, Hailman E, Johns DG, Hubbard BK, Yee LP, Wright SD, Desouza MM, Plump A, Reiser V (2011). A gene expression signature that classifies human atherosclerotic plaque by relative inflammation status. Circ Cardiovasc Genet.

[62] Huang J, Hu W, Hu L, Previs RA, Dalton HJ, Yang XY, Sun Y, McGuire M, Rupaimoole R, Nagaraja AS, Kang Y, Liu T, Nick AM, Jennings NB, Coleman RL, Jaffe RB, Sood AK (2016). Dll4 Inhibition plus Aflibercept markedly reduces ovarian tumor growth. Mol Cancer Ther.

[63] Stather PW, Sylvius N, Sidloff DA, Dattani N, Verissimo A, Wild JB, Butt HZ, Choke E, Sayers RD, Bown MJ (2015). Identification of microRNAs associated with abdominal aortic aneurysms and peripheral arterial disease. Br J Surg.

