# Supplementary material for: Distinct gene expression profiles associated with Notch ligands Delta-like 4 and Jagged1 in plaque material from peripheral artery disease patients: a pilot study
Source: J Transl Med. 2017 May 4;15:98. doi: 10.1186/s12967-017-1199-3 (PMC5418727; doi:10.1186/s12967-017-1199-3)
Supplement: Supplementary file 1 — Additional file 1: Figure S1. Characterization of primary cultures of rat aortic smooth muscle cells by immunofluorescent staining. Figure S2. Oil-red staining of cholesterol loaded rat aortic smooth muscle cells. Table S1. Primers used for quantitative RT-PCR. Table S2. Semiquantitative evaluation of histological characteristics and Notch receptors immunostainings. Table S3. P values associated to the results of correlations analyses among the expression levels of mRNAs and miRs analyzed. Table S4. Clinical follow up and molecular analysis. [file 12967_2017_1199_MOESM1_ESM.doc]

**ADDITIONAL FILE 1**

**Fig. S1**: **Characterization of primary cultures of rat aortic smooth muscle cells by immunofluorescent staining**

**
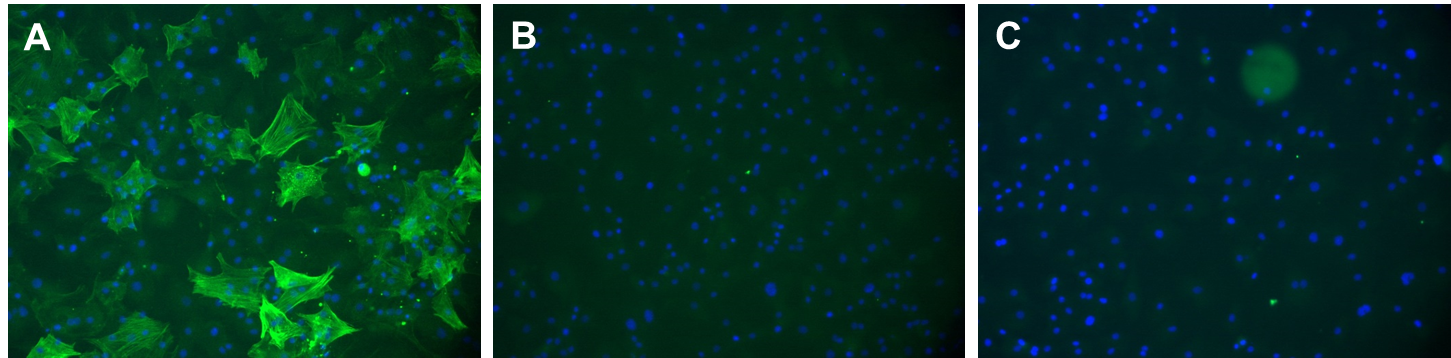
**

Representative images of RASMCs immunolabelled with α-SM-actin (A) and VE-cadherin (B) antibodies. (C) Negative Isotype IgG control. DAPI staining (blue) was used to visualize nuclei. (40X objective).

**Fig. S2: Oil-red staining of cholesterol loaded rat aortic smooth muscle cells**

**
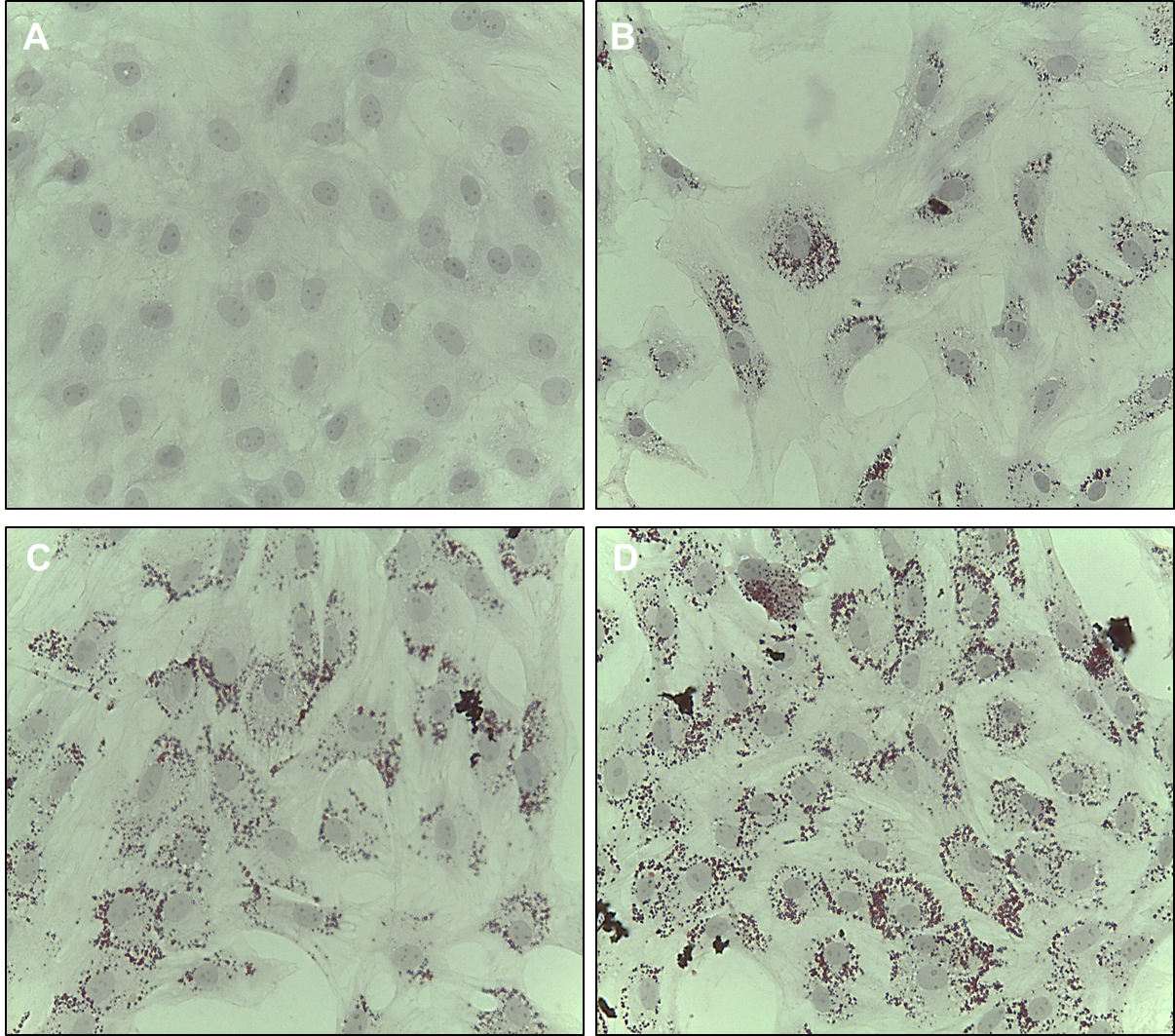
**

Representative images of RASMCs with Oil Red O after loading with 20 (B), 50 (C) and 100 (D) μg/ml of cholesterol for 72 hours. (A) Cells grown in control medium. (20X objective).

**Table S1 Primers used for quantitative** RT-PCR

|  | **HOMO SAPIENS PRIMER SEQUENCE (5’-3’)** | **RATTUS NORVEGICUS PRIMER SEQUENCE (5’-3’)** |
| --- | --- | --- |
| **RPL13A F** | GGAGGTGCAGGTCCTGGTGCTT | CCGCAAGATCCGCAGACGCA |
| **RPL13A R** | CGTACGACCACCACCTTCCGG | CTGATGGGACCGGACGCGG |
| **HEY1 F** | CCGAGATCCTGCAGATGACCGT |  |
| **HEY1 R** | AACGCGCAACTTCTGCCAGG |  |
| **HEY2 F** | AAAAGGCGTCGGGATCG | TTTCGCCGCGATGAAGCGCC |
| **HEY2 R** | AGCTTTTTCTAACTTTGCAGATCC | TGAGCTTGCACTGTGCCCGGAG |
| **HES1 F** | CGGACATTCTGGAAATGACA | ATTCCTCGTCCCCGGTGGCT |
| **HES1 R** | CATTGATCTGGGTCATGCAG | TCTTGCCCGGCGCCTCTTCT |
| **DLL4 F** | GCGAGAAGAAAGTGGACAGG | ACGACCTGCGGCCAGAGACT |
| **DLL4 R** | ATTCTCCAGGTCATGGCAAG | CGCTGCAGACGACCCGGTAA |
| **JAGGED-1 F** | GACTCATCAGCCGTGTCTCA | ATGCCTCCTGTCGGGATTTG |
| **JAGGED-1 R** | TGGGGAACACTCACACTCAA | AGTGACCCCCATTCAAGCAG |
| **HEYL F** | CAAGAGGGCCAGCTGAGCCAGA |  |
| **HEYL R** | GCGGTCTCGACGCCGTTTCT |  |
| **SM22 F** | GAATTGATGGAAACCACCGGG | CATCACCAACTGGGACGACA |
| **SM22 R** | GGGAAAGCTCCTTGGAAGTTCT | TCCGTTAGCAAGGTCGGATG |
| **VCAM-1 F** | GGTATCTGCATCGGGCCTC |  |
| **VCAM-1 R** | TAAAAGCTTGAGAAGCTGCAAACA |  |
| **CD68 F** | CACAGTGGACATTCTCGGCT | ACGGACAGCTTACCTTTGGA |
| **CD68 R** | GGTGGACAGCTGGTGAAAGA | GAATGTCCACTGTGCTGCTTG |
| **COX2 F** | CAAATTGCTGGCAGGGTTGC |  |
| **COX2 R** | AGGGCTTCAGCATAAAGCGT |  |
| **Bcl2 F** | CATGCTGGGGCCGTACAG |  |
| **Bcl2 R** | GAACCGGCACCTGCACAC |  |
| **HMGCR F** |  | TGCAGAGAAAGGTGCGAAGT |
| **HMGCR R** |  | TGCGTCTCCATGAGGGTTTC |
| **MCP-1 F** |  | TTAATGCCCCACTCACCTGC |
| **MCP-1 R** |  | GAGCTTGGTGACAAATACTACAGC |

List of primers used for quantitative RT-PCR analysis of mRNA isolated from patients’ plaque material (HOMO SAPIENS PRIMER SEQUENCE) and rat aortic smooth muscle cells (RATTUS NORVEGICUS PRIMER SEQUENCE).

**Table S2 Semiquantitative evaluation of histological characteristics and Notch receptors** immunostainings

|  | **HISTOLOGICAL CHARACTERISTICS** | | | | | | **NOTCH (IHC)** | | |
| --- | --- | --- | --- | --- | --- | --- | --- | --- | --- |
| **Ptz ID** | **Cellularity** | **Loose**  **fibrous tissue** | **Dense**  **fibrous tissue** | **Calcification** | **Lipids** | **Inflammatory**  **infiltrate** | **Notch1** | **N1IC** | **Notch3** |
| **2** | 1 | 1 | 1 | 1 | 1 | 1 | 2 | 1 | 3 |
| **4** | 2 | 3 | 3 | 2 | 3 | 2 | 2 | 1 | 3 |
| **5** | 2 | 1 | 3 | 2 | 2 | 1 | 3 | 3 | 3 |
| **8** | 3 | 1 | 3 | 1 | 2 | 2 | 3 | 3 | 3 |
| **10** | 3 | 1 | 3 | 1 | 2 | 1 | 3 | 2 | 3 |
| **11** | 3 | 1 | 3 | 1 | 2 | 1 | 3 | 1 | 3 |
| **12** | 2 | 1 | 3 | 2 | 2 | 2 | 3 | 1 | 3 |
| **13** | 3 | 2 | 2 | 3 | 3 | 1 | 1 | 1 | 3 |
| **14** | 2 | 1 | 2 | 1 | 1 | 2 | 2 | 1 | 2 |
| **15** | 3 | 3 | 3 | 1 | 1 | 2 | 2 | 0 | 2 |
| **16** | 3 | 3 | 3 | 1 | 2 | 3 | 2 | 1 | 3 |
| **17** | 1 | 1 | 3 | 1 | 1 | 1 | N/A | N/A | N/A |
| **18** | 2 | 2 | 2 | 1 | 2 | 2 | 1 | 0 | 3 |
| **19** | 3 | 2 | 3 | 1 | 3 | 3 | 3 | 2 | 3 |
| **20** | 2 | 1 | 3 | 1 | 2 | 3 | 2 | 1 | 2 |
| **21** | 1 | 1 | 3 | 1 | 1 | 1 | N/A | N/A | N/A |
| **22** | 3 | 1 | 3 | 1 | 2 | 1 | 3 | 1 | 3 |
| **23** | 2 | 1 | 3 | 1 | 1 | 1 | 3 | 3 | 3 |
| **24** | 2 | 1 | 3 | 1 | 1 | 1 | 3 | 3 | 3 |
| **25** | 3 | 3 | 3 | 1 | 2 | 1 | 2 | 2 | 3 |

Criteria for grading criteria are provided in Material and Methods-On line. 1 (low), 2 (medium) and 3 (high) is referred to the number of cells (cellularity, inflammatory infiltrate, IHC) or to the percent of stained area (loose fibrous tissue, dense fibrous tissue, calcification and lipids) in patients’ plaque material. Abbreviations: IHC – Immunohistochemistry; N1IC – Intracellular Notch1; N/A – Not Available; Ptz ID – Patient Identification.

**Table S3 p values associated to the results of correlations analyses among the expression levels of mRNAs and miRs analyzed**

| **Gene** | **DLL4** | **Jag1** | **Hey1** | **Hey2** | **COX2** | **VCAM** | **CD68** | **sm22** | **Bcl2** | **HeyL** | **Hes1** | **miR155** | **miR125** | **miR126** | **miR146** | **miR424** | **miR21** |
| --- | --- | --- | --- | --- | --- | --- | --- | --- | --- | --- | --- | --- | --- | --- | --- | --- | --- |
| **DLL4** | 0 | 0,059 | 0,948 | **0,021** | **0,008** | 0,050 | 0,359 | **0,011** | **0,037** | 0,097 | 0,108 | 0,270 | **0,025** | 0,050 | 0,133 | **0,011** | 0,581 |
| **Jag1** | 0,059 | 0 | 0,678 | **0,025** | 0,097 | 0,194 | 0,059 | **0,002** | **0,021** | **0,001** | 0,613 | **0,005** | 0,250 | 0,076 | 0,148 | 0,097 | 0,708 |
| **Hey1** | 0,948 | 0,678 | 0 | 0,776 | 0,880 | 0,336 | 0,880 | 0,521 | 0,270 | 0,744 | 0,336 | 0,843 | 0,552 | 0,912 | 0,521 | 0,948 | 0,213 |
| **Hey2** | **0,021** | **0,025** | 0,776 | 0 | **0,002** | **0,014** | 0,086 | **0,006** | 0,108 | **0,006** | 0,313 | **0,021** | 0,059 | **0,014** | 0,213 | 0,108 | 0,708 |
| **COX2** | **0,008** | 0,097 | 0,880 | **0,002** | 0 | **0,031** | 0,270 | **0,014** | 0,076 | **0,037** | 0,270 | 0,194 | 0,059 | 0,067 | 0,463 | 0,097 | 0,744 |
| **VCAM** | 0,050 | 0,194 | 0,336 | **0,014** | **0,031** | 0 | **0,043** | 0,050 | 0,708 | 0,108 | 0,213 | 0,086 | **0,011** | **0,043** | **0,037** | 0,059 | 0,178 |
| **CD68** | 0,359 | 0,059 | 0,880 | 0,086 | 0,270 | **0,043** | 0 | **0,031** | 0,581 | 0,050 | 0,678 | **0,006** | 0,194 | **0,031** | **0,031** | 0,148 | 0,744 |
| **sm22** | **0,011** | **0,002** | 0,521 | **0,006** | **0,014** | 0,050 | **0,031** | 0 | **0,031** | **0,005** | 0,493 | **0,021** | 0,086 | **0,011** | 0,097 | **0,017** | 0,880 |
| **Bcl2** | **0,037** | **0,021** | 0,270 | 0,108 | 0,076 | 0,708 | 0,581 | **0,031** | 0 | 0,067 | 0,336 | 0,270 | 0,463 | 0,178 | 0,708 | 0,194 | 0,336 |
| **HeyL** | 0,097 | **0,001** | 0,744 | **0,006** | **0,037** | 0,108 | 0,050 | **0,005** | 0,067 | 0 | 0,644 | **0,003** | 0,133 | 0,086 | 0,213 | 0,270 | 0,581 |
| **Hes1** | 0,108 | 0,613 | 0,336 | 0,313 | 0,270 | 0,213 | 0,678 | 0,493 | 0,336 | 0,644 | 0 | 0,744 | 0,059 | 0,313 | 0,194 | 0,463 | 0,880 |
| **miR-155** | 0,270 | **0,005** | 0,843 | **0,021** | 0,194 | 0,086 | **0,006** | **0,021** | 0,270 | **0,003** | 0,744 | 0 | 0,270 | 0,059 | 0,097 | 0,250 | 0,982 |
| **miR125a** | **0,025** | 0,250 | 0,552 | 0,059 | 0,059 | **0,011** | 0,194 | 0,086 | 0,463 | 0,133 | 0,059 | 0,270 | 0 | 0,086 | **0,021** | 0,178 | 0,744 |
| **miR126** | 0,050 | 0,076 | 0,912 | **0,014** | 0,067 | **0,043** | **0,031** | **0,011** | 0,178 | 0,086 | 0,313 | 0,059 | 0,086 | 0 | 0,059 | **0,043** | 0,552 |
| **miR146** | 0,133 | 0,148 | 0,521 | 0,213 | 0,463 | **0,037** | **0,031** | 0,097 | 0,708 | 0,213 | 0,194 | 0,097 | **0,021** | 0,059 | 0 | 0,086 | 0,521 |
| **miR424** | **0,011** | 0,097 | 0,948 | 0,108 | 0,097 | 0,059 | 0,148 | **0,017** | 0,194 | 0,270 | 0,463 | 0,250 | 0,178 | **0,043** | 0,086 | 0 | 0,133 |
| **miR21** | 0,581 | 0,708 | 0,213 | 0,708 | 0,744 | 0,178 | 0,744 | 0,880 | 0,336 | 0,581 | 0,880 | 0,982 | 0,744 | 0,552 | 0,521 | 0,133 | 0 |

Bold characters highlight significant p values (< 0.05**).**

**Table S4 Clinical follow up and molecular analysis**

| **Ptz ID** |  | **Molecular analysis** | | | | | **Clinical Follow-Up** | |
| --- | --- | --- | --- | --- | --- | --- | --- | --- |
|  |  | **GEP** | | **DLL4** | **COX2** | | **6 months** | **12 months** |
| **2** |  |  |  |  |  |  | no ischemic events | claudication started at the contralateral leg |
| **4** |  |  |  |  |  |  | no ischemic events | claudication started at the contralateral leg |
|  |  |  |  |  |  |  | new symptoms at the other leg |  |
| **5** |  |  |  |  |  |  | no ischemic events | no ischemic events |
| **8** |  | S |  | LOW |  | LOW | no ischemic events | no ischemic events |
| **10** |  |  |  |  |  |  | coronary event | coronary event |
| **11** |  |  |  |  |  |  | N/A | N/A |
| **12** |  | M |  | LOW |  | HIGH | claudication at the treated leg | N/A |
|  |  |  |  |  |  |  | new symptoms at the other leg |  |
| **13** |  |  |  |  |  |  | no ischemic events | coronary event |
| **14** |  | IF |  | HIGH |  | HIGH | N/A | N/A |
| **15** |  | M |  | LOW |  | LOW | no ischemic events | no ischemic events |
| **16** |  | M |  | HIGH |  | HIGH | claudication at the treated leg | symptoms recurred at the same leg |
| **17** |  |  |  |  |  |  | no ischemic events | no ischemic events |
| **18** |  |  |  |  |  |  | N/A | N/A |
| **19** |  | M |  | LOW |  | LOW | no ischemic events | no ischemic events |
| **20** |  | IF |  | HIGH |  | HIGH | new symptoms at the other leg | symptoms recurred at the same leg |
|  |  |  |  |  |  |  | symptoms recurred at the same leg | coronary event |
| **21** |  |  |  |  |  |  | coronary event | no ischemic events |
| **22** |  |  |  |  |  |  | no ischemic events | symptoms recurred at the same leg |
|  |  |  |  |  |  |  | claudication at the treated leg |  |
| **23** |  | S |  | LOW |  | LOW | coronary event | no ischemic events |
| **24** |  |  |  |  |  |  | aneurism | no ischemic events |
| **25** |  | S |  | LOW |  | LOW | no ischemic events | no ischemic events |

Highlighted in red (symptomatic) or in green (asymptomatic) are those patients for which both clinical follow-up and molecular analysis were available. Abbreviation: S - stable plaque profile; M - mixed plaque profile; IF - inflamed plaque profile; GEP – Gene expression profile; N/A – Not available.
